# Characterisation of progression of macular oedema in the initial stages of diabetic retinopathy: a 3-year longitudinal study

**DOI:** 10.1038/s41433-022-01937-3

**Published:** 2022-01-22

**Authors:** Conceição Lobo, Torcato Santos, Inês P. Marques, Maria H. Madeira, Ana Rita Santos, João Figueira, José Cunha-Vaz

**Affiliations:** 1 AIBILI - Association for Innovation and Biomedical Research on Light and Image, 3000-548 Coimbra, Portugal; 2 University of Coimbra, Coimbra Institute for Clinical and Biomedical Research (iCBR), Faculty of Medicine, 3000-548 Coimbra, Portugal; 3 Center for Innovative Biomedicine and Biotechnology (CIBB), University of Coimbra, 3000-548 Coimbra, Portugal; 4 Department of Ophthalmology, Centro Hospitalar e Universitário de Coimbra (CHUC), 3000-548 Coimbra, Portugal; 5 Department of Orthoptics, School of Health, Polytechnic of Porto, Porto, Portugal

**Keywords:** Prognostic markers, Predictive markers

## Abstract

**Background/objectives:**

To characterise the prevalence and three-year progression of centre-involving diabetic macular oedema (CI-DMO) in minimal to moderate non-proliferative diabetic retinopathy, using optical coherence tomography (OCT) and measurements of retinal fluid using tissue optical reflectivity ratios (OCT-Leakage).

**Methods/methods:**

Seventy-four eyes from 74 patients were followed in a 3-year prospective longitudinal observational cohort of type 2 diabetes (T2D) patients using spectral-domain optical coherence tomography (SD-OCT), OCT-Angiography (OCT-A) and OCT-Leakage (OCT-L). Eyes were examined four times with 1-year intervals. Sixteen eyes (17.8%) were excluded from the analysis due to quality control standards. Retinal oedema was measured by central retinal thickness and retinal fluid by using optical reflectivity ratios obtained with the OCT-L algorithm. Vessel density was measured by OCT-A. Thinning of the ganglion cell and inner plexiform layers (GCL + IPL) was examined to identify retinal neurodegenerative changes. Diabetic retinopathy ETDRS classification was performed using the seven-field ETDRS protocol.

**Results:**

CI-DMO was identified in the first visit in 9% of eyes in ETDRS groups 10–20, 10% of eyes in ETDRS group 35 and 15% of eyes in ETDRS groups 43–47. The eyes with CI-DMO and subclinical CI-DMO showed a progressive increase in retinal extracellular fluid during the 3-year period of follow-up. The eyes with CI-DMO and increased retinal extracellular fluid accumulation were associated with vision loss.

**Conclusions:**

The prevalence of subclinical CI-DMO and CI-DMO in the initial stages of NPDR occurs independently of severity grading of the retinopathy, showing progressive increase in retinal extracellular fluid and this increase is associated with vision loss (82% 9 out of 11 cases).

## Introduction

Macular oedema is the most frequent cause of vision loss due to diabetic retinopathy [1]. Its characterisation and understanding of its progression are fundamental for the development of more timely and targeted treatments. Optical Coherence Tomography (OCT) allows objective and quantitative evaluation of retinal oedema, providing information on the microstructure of the retina and reproducible thickness measurements. Using OCT images, an algorithm designated as OCT-Leakage (OCT-L) is capable of performing automated analyses of the abnormal retinal extracellular space by identifying and measuring sites of lower optical reflectivity [2–4].

Using these imaging tools, OCT, OCT-Angiography (OCT-A) and OCT-L, we examined eyes in the initial stages of non-proliferative diabetic retinopathy (NPDR), identified by the Early Treatment Diabetic Retinopathy Study (ETDRS) grading, for a period of three years with annual examinations, to study the rates of occurrence of macular oedema, considering the categories of centre-involving diabetic macular oedema (CI-DMO) and subclinical CI-DMO with and without visual loss [5–7]. Centre-involving diabetic macular oedema and subclinical CI-DMO were defined using objective and predefined values of central retinal thickness (CRT), proposed in DRCR.net studies [5, 6].

## Materials and methods

The study was designed to analyse eyes with type 2 diabetes (T2D) and with NPDR (ETDRS grades 10–47), which have completed the 3-year follow-up with annual examinations with OCT and OCT-Angiography (OCT-A). The tenets of the Declaration of Helsinki were followed, approval was obtained from the Institutional Ethical Review Board and written informed consent to participate in the study was obtained from all individuals after all procedures were explained.

Exclusion criteria included any previous laser treatment or intravitreal injections, presence of other retinal diseases (e.g. age-related macular degeneration, glaucoma, or vitreomacular disease), high ametropia (spherical equivalent greater than –6 and +2 dioptres), or any other systemic disease that could affect the eye, with special attention for uncontrolled systemic hypertension (values outside normal range: systolic 70–210 mmHg and diastolic 50–120 mmHg) and history of ischaemic heart disease. Cataract surgery was another exclusion criteria.

A total of 90 eyes from 90 patients with confirmed T2D and ETDRS levels between 10 and 47 were included, with a maximum glycated haemoglobin A_1C_ (HbA_1C_) value of 10%. An age-matched population including 84 eyes of individuals without diabetes or other retinal diseases was used as control group to set normal values and identify abnormal deviations between diabetic and non-diabetic populations. No follow-up of non-diabetic individuals was performed. Age, duration of diabetes, HbA1c, and blood pressure levels were collected for each participant at baseline visit.

All participants underwent full ophthalmological examination, including visual acuity, 7-field colour fundus photography (CFP), SD-OCT, and OCT-A imaging, in intervals of 1 year (baseline, 1-year, 2-year and 3-year visits).

### Seven-field colour fundus photography

The seven-fields CFP were acquired using the Topcon TRC 50DX camera (Topcon Medical Systems, Tokyo, Japan), at 30°/35°. The diabetic retinopathy (DR) severity score was determined at baseline and every annual visit by two independent graders in a context of an experienced reading centre (Coimbra Ophthalmology Reading Center—CORC, Coimbra, Portugal) using a modified Airlie House classification scheme according to the ETDRS Protocol [8, 9]. Step changes in the ETDRS retinopathy severity scale were used to describe worsening or improvement of the retinopathy [10, 11].

### Best-corrected visual acuity evaluation

Best-corrected visual acuity (BCVA) was evaluated and recorded as letters read at 4 m on ETDRS charts. Final BCVA letter score was calculated by adding the number of letters read at 4 m plus 30 (or the number of letters read at 1 m). BCVA was converted into logarithm units of the minimal angle of resolution [12]. The presence of any visual loss was recorded.

### Optical coherence tomography

OCT was performed using the Cirrus HD-OCT 5000 (Carl Zeiss Meditec, Inc., Dublin, CA, USA). The Macular Cube 512 × 128 acquisition protocol, consisting of 128 B-scans with 512 A-scans each, was used to assess the subjects’ CRT and the thickness of the Ganglion Cell Layer and Inner Plexiform Layer (GCL + IPL), both automatically calculated by the equipment. Neurodegeneration (ND) was defined as GCL + IPL thinning larger than 2 standard deviations (SD) of the healthy control population. CRT was defined as the retinal thickness of the central subfield (1 mm diameter circle centred on the fovea) and the GCL and IPL thickness is the average layer thickness in the inner ring (1 mm to 3 mm diameter annulus centred on the fovea). These measurements were performed using the manufacture’s software available in the equipment and software parameters are not available for scientific scrutiny to the best of our knowledge. Results with different devices are not directly comparable.

Central retinal thickness increases were used to identify macular oedema. Subclinical CI-DMO and CI-DMO were defined by objective SD-OCT measurements following a set of predefined, gender-dependent, rules based on previous publications of the DRCR.net and for the Zeiss SD-OCT devices [6]. Age was not taken into consideration for the definition. For male subjects, eyes with CRT greater than 290 µm and less than 305 µm (CRT ≥ 1SD) were classified as having subclinical CI-DMO, and eyes with CRT greater than 305 µm (CRT ≥ 2SD) classified as having CI-DMO. For female subjects, CRT greater than 260 µm were classified as subclinical CI-DMO (CRT ≥ 1SD) while CRT values greater than 290 µm were classified as CI-DMO (CRT ≥ 2SD).

### OCT-Leakage

The OCT-Leakage algorithm is an image analysis algorithm based on the projection of low optical reflectivity (LOR) voxels of the structural OCT data to a plane perpendicular to the depth direction. LOR voxels are identified by thresholding the reflectivity intensity values from the structural OCT data by a reference value calculated from a normative database of healthy control subjects. White areas depicted in the LOR maps represent the locations of A-scans having reflectivity values less than the predefined threshold; otherwise, locations appear as black areas. Extracellular fluid distribution of a given area of the retina can be measured by the LOR area ratio, which stands for the fraction of the number of A-scans with optical reflectivity values less than the threshold and the total number of A-scans within the considered area. LOR sites were identified in the different retinal layers. Maps of LOR sites and LOR area ratios values are therefore obtained not only for the full retina, but also layer by layer as *en-face* images [2–4]. Fluid accumulation of higher density such as exudates is underestimated.

Fluctuations of extracellular fluid accumulation in the retina were assessed by the variation in LOR values through the follow-up visits. A CI-DMO or subclinical CI-DMO eye was identified as having LOR fluctuations if they presented variations in LOR values greater than 1SD of the normal distribution of LOR values for all eyes with increased CRT.

The OCT-L algorithm was applied to the structural OCT data acquired in the central macular 6 × 6 mm^2^ area with the Cirrus HD-OCT 5000. The acquisition protocol consists of 350 B-Scans, each composed of 350 A-Scans evenly spread through the scanned area of the central macula.

### OCT-Angiography

OCT-A data were collected by the Cirrus HD-OCT 5000 device using the Angiography 3 × 3-mm^2^ acquisition protocol, which consists of a set of 245 clusters of 4 B-Scans repetitions, where each B-Scan comprises 245 A-Scans, over a 3 × 3 × 2 mm^3^ volume in the central macula.

The Carl Zeiss Meditec Density Exerciser (version:10.0.0.12787) was applied to calculate vessel density (VD) and foveal avascular zone (FAZ) [13]. Vessel Density for the inner ring region was analysed for the Superficial capillary plexus (SCP), Deep capillary plexus (DCP) and full retina (FR). Circularity index of the FAZ detected on the SCP was also computed. Finally, normalisation for signal strength was performed [14]. Vessel closure was identified by decreased VD metrics measured in the SCP, DCP, and FR.

### Image quality assessment

OCT-A and OCT-L data were reviewed for image quality assessment. All examinations underwent a quality check to discard acquisitions having a signal strength lower than 7. In addition, OCT-A were also reviewed for motion artifacts or evidence of defocus or blur. The acquisition was classified as low quality if artifacts were present in more than 25% of the area under analysis. If one of the low-quality criteria occurred in the first or last follow-up visit, the patient was discarded from the analysis, otherwise only the low-quality acquisition data was discarded.

**Fig. 1:**
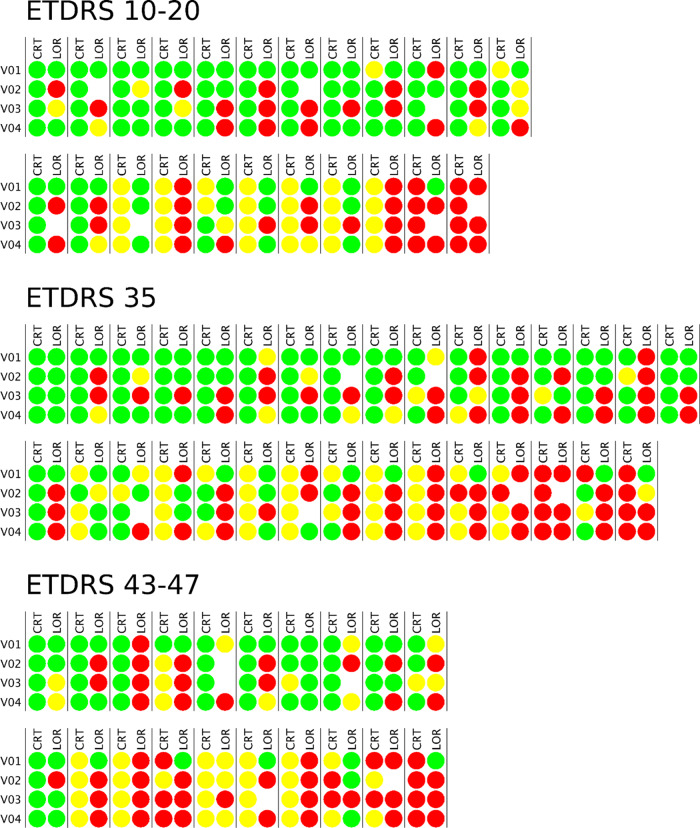
Schematic representation of individual central retinal thickness and low optical reflectivity ratio at the Central Subfield and its progression over the four visits. The values are given in relation to the control group: Values within a normal range are depicted in green; 2SD increase is depicted in red; 1SD increase is depicted in yellow. Empty spaces indicate that reliable measurements could not be obtained in that specific visit due to insufficient image quality. CRT: central retinal thickness; LOR: low optical reflectivity.

From 90 eyes enroled in this study, 17.8% were excluded as they did not meet the set of quality criteria in the first or last visits, leading to a final number of 74 eyes that allowed analysis of the 3-year progression (Fig. 1).

### Statistical analysis

Variables were summarised for each ETDRS group, 10–20, 35, and 43–47, using mean and SD.

The χ^2^ test for categorical variables and the Kruskal-Wallis-H test for continuous variables were performed for comparison between the three ETDRS groups. To assess statistically significant differences between the measurements of healthy controls and each ETDRS group, the χ^2^ test was used for categorical variables and the Mann-Whitney *U* test was used for continuous variables.

For comparison of the progression of the disease in terms of VD and GCL + IPL layer, Mann-Whitney *U* tests were used. Correlation between changes of CRT and LOR from the first to the last visits was assessed by Spearman’s rank correlation coefficient. Statistical analysis was performed with Stata 16.1 (StataCorp LLC), and a *p* value ≤ 0.05 was considered statistically significant.

## Results

Of the 74 eyes analysed, 23 (31%) were graded as ETDRS levels 10–20 at baseline, 31 (42%) as ETDRS level 35 and 20 as ETDRS levels 43–47 (27%). The observed agreement between the 2 graders was 97%. All disagreement cases were resolved by mutual agreement [15]. Demographic and baseline systemic and ocular parameters of these 74 T2D eyes and 84 age-matched healthy controls included in the study are presented in Table 1. Of the systemic variables, only HbA1C correlates positively with DR severity (*p* = 0.008) (Table 1).

**Table 1:** Baseline characteristics for healthy controls and T2D individuals considering distinct ETDRS groups.

	Healthy controls	ETDRS 10–20	ETDRS 35	ETDRS 43–47	*p* value (between the three ETDRS groups)^b^
	(*n* = 84)	(*n* = 23)	(*n* = 31)	(*n* = 20)	
Sex, Male/female	39/45	15/8	25/6	16/4	0.499
		*0.08*	**0.001**	**0.019**	
Age, years	69.2 ± 4.5	69.6 ± 6.0	65.4 ± 5.5	65.6 ± 7.2	0.064
		0.706	**0.002**	0.213	
Diabetes duration, years	---	18.3 ± 7.3	16.7 ± 6.5	17.2 ± 4.8	0.766
BCVA, letters	---	82.5 ± 4.2	82.7 ± 4.6	81.0 ± 8.2	0.672
HbA1c, %	---	7.0 ± 1.1	7.3 ± 1.1	8.0 ± 1.2	**0.008**
LOR INL, CSF, µm (40 healthy controls) (*p*-value^a^)	0.015 ± 0.008	0.018 ± 0.014	0.021 ± 0.017	0.023 ± 0.015	0.353
		0.803	0.345	**0.032**	
RT, CSF, µm (58 healthy controls) (*p*-value^a^)	260.6 ± 18.3	265.2 ± 27.3	270.1 ± 27.1	274.1 ± 23.8	0.549
		0.393	0.162	**0.034**	
GCL + IPL, Inner Ring, µm (58 healthy controls) (*p*-value^a^)	82.7 ± 5.5	83.0 ± 9.3	77.6 ± 8.1	78.5 ± 6.8	0.098
		0.846	**0.003**	**0.018**	
VD,SCP, inner ring, mm^−1^ (*p*-value^a^)	22.3 ± 0.9	21.8 ± 1.2	20.9 ± 1.1	21.2 ± 1.3	**0.023**
		0.098	**<0.001**	**<0.001**	
VD,DCP, inner ring, mm^−1^ (*p*-value^a^)	17.0 ± 2.1	17.1 ± 1.97	16.2 ± 2.2	16.8 ± 2.0	0.279
		0.879	0.078	0.792	
VD,FR, inner ring, mm^−1^ (*p*-value^a^)	23.7 ± 0.9	23.6 ± 1.1	22.6 ± 1.1	22.9 ± 1.2	**0.007**
		0.850	***p*** < **0.001**	**0.030**	
FAZ circularity index (*p*-value^a^)	0.69 ± 0.07	0.64 ± 0.16	0.61 ± 0.14	0.57 ± 0.09	**0.014**
		0.636	0.205	***p*** < **0.001**	

Data presented as Mean ± SD.

*BCVA* best-corrected visual acuity, *HbA1c* glycated haemoglobin, *LOR* low optical reflectivity, *INL* inner nuclear layer, *RT* retinal thickness, *CSF* central sub-field, *GCL + IPL* ganglion cell and inner plexiform layers, *VD* vessel density, *SCP* superficial capillary plexus, *DCP* deep capillary plexus, *FR* full retina, *FAZ* foveal avascular zone.

Bold values indicate statistical significance *p* < 0.05.

Data was not collected for dashed table entries.

^a^X² test (categorical variables) and Mann-Whitney *U*-test (continuous variables) for comparison between Healthy Controls and each ETDRS group.

^b^X² test (categorical variables) and Kruskal Wallis test (continuous variables) for comparison between the three ETDRS groups.

The changes in CRT did not show correlation with progression in the severity of retinopathy (Table 2 and Fig. 1). Only VD showed progressively decreasing values (*p* = 0.03) whereas thinning of the GCL + IPL representing ND remained in the same range during the 3-year follow-up.

**Table 2:** Central retinal thickness (oedema), vessel density (capillary closure) and ganglion cell and inner plexiform layers thinning (neurodegeneration) comparison between T2D individuals that worsened 1- or 2-steps in DRSS stage vs the ones that improved or did not change, at the end of the follow-up period.

		CRT [µm]		VD (Inner Ring SCP) [mm^−1^]		GCL + IPL Thinning [µm]	
		V1	V4-V1	V1	V4-V1	V1	V4-V1
Worsening (1- or 2-step) (*n* = 10)	Avg	254.5	1.6	21.5	−1.2	82.3	−0.7
	SD	26.9	3.6	0.9	1.0	9.0	1.2
No Change + Improving (*n* = 64)	Avg	272.0	1.7	21.2	−0.4	79.1	−1.4
	SD	25.4	17.5	1.3	1.0	8.3	5.2
Statistical Diff. V4-V1 (*p* value)^a^			0.61		** 0.03**		0.94

*CRT* central retinal thickness, *VD* vessel density, *SCP* superficial capillary plexus, *GPL* *+* *IPL* ganglion cell layer and inner plexiform layer.

^a^Mann-Whitney U-test for comparison between DRSS stage worsening (1- or 2- step) and improving or no change patients. Bold value represents statistical significance (*p*  <  0.05).

At baseline, when considering CRT values, there were 25 eyes from 74 eyes/patients (34%) with subclinical CI-DMO and 8 eyes with CI-DMO (11%). The eyes with CI-DMO were distributed similarly in the three ETDRS groups, 10–20 (9%), 35 (10%) and 43-47 (15%) (Table 3). At the end of the three-year period of follow-up the prevalence of CI-DMO remained stable with 9% (ETDRS 10–20), 7% (ETDRS 35) and 10% (ETDRS 43–47). The prevalence of subclinical CI-DMO at baseline was also similar between different ETDRS groups with 39% for ETDRS grades 10–20, 32% for grade 35 and 30% for grades 43–47. At the last visit they remained stable with 26% (ETDRS 10–20), 32% (ETDRS 35) and 40% (ETDRS 43–47) (Table 3).

**Table 3:** Number and percentage of eyes with subclinical CI-DMO and CI-DMO by ETDRS group at baseline (V1) and at the 3-year follow-up visit (V4) and respective LOR fluctuations and BCVA variations.

	Subclinical CI-DMO (CRT ≥ 1 SD and <2SD)				CI-DMO (CRT ≥ 2 SD)				
	ETDRS 10–20	ETDRS 35		ETDRS 43–47	ETDRS 10-20		ETDRS 35		ETDRS 43–47
Baseline (V1)	9/23 (39%)	10/31 (32%)		6/20 (30%)	2/23 (9%)		3/31 (10%)		3/20 (15.0%)
3-year Visit (V4)	6/23 (26%)	10/31 (32%)		8/20 (40%)	2/23 (9%)		2/31 (7%)		2/20 (10.0%)
Number of eyes with increased LOR ratio (≥1SD)									
	Subclinical CI-DMO				CI-DMO				
	ETDRS 10–20	ETDRS 35		ETDRS 43–47	ETDRS 10–20		ETDRS 35		ETDRS 43–47
Baseline (V1)	2/9 (22%)	4/10 (40%)		3/6 (50%)	1/2 (50%)		1/3 (33%)		1/3 (33%)
3-year Visit (V4)	3/6 (50%)	7/10 (70%)		7/8 (88%)	2/2 (100%)		2/2 (100%)		2/2 (100%)
LOR fluctuations and BCVA variations over the follow-up period of subclinical CI-DMO or CI-DMO eyes									
			Vision Gain			No Vision Change		Vision Loss	
Without LOR fluctuations			8 (67%)			2 (29%)		2 (18%)	
With LOR fluctuations			4 (33%)			5 (71%)		9 (82%)	
			12			7		11	

*CI-DMO* centre-involving diabetic macular oedema, *CRT* central retinal thickness, *SD* standard deviation, *LOR* low optical reflectivity, *BCVA* best-corrected visual acuity.

In the period of three years of follow-up no eyes with subclinical CI-DMO at baseline progressed to CI-DMO. Similarly, the eyes that were identified with CI-DMO at baseline either remained with CI-DMO or showed a decrease in CRT presenting subclinical CI-DMO after three years of follow up. Association with visual loss during the follow-up was present in three of the six eyes with CI-DMO at the final visit.

**Fig. 2:**
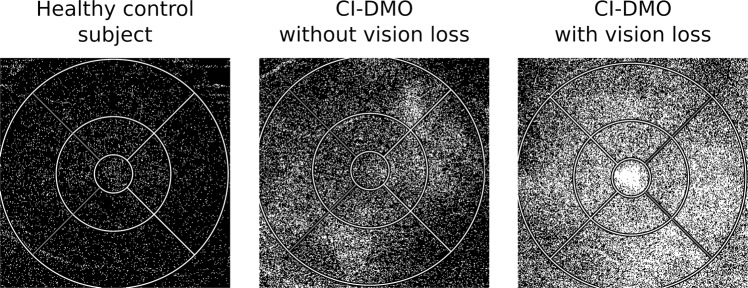
OCT-Leakage maps of a healthy control and of CI-DMO eyes with and without vision loss at the 3-year follow-up visit. OCT-Leakage LOR map for the full retina of a healthy control subject (left), a CI-DMO patient without vision loss (centre) and CI-DMO patient with vision loss (right) at the end of the follow-up period. White areas represent areas of increased extracellular space. CI-DMO: Central-involved diabetic macular oedema; LOR: Low Optical Reflectivity.

When examining the LOR ratio values, it was possible to identify an increase in the retinal extracellular fluid accumulation during the three-year period of follow-up (Fig. 2). There was an increase in the LOR ratio values in the eyes with subclinical CI-DMO from 22% to 50% in grades ETDRS 10-20, from 40% to 70% in grade ETDRS 35 and from 50% to 88% in grades ETDRS 43–47. An increase in the LOR ratio values was also identified during the 3-year period in all eyes with CI-DMO from 50% to 100% (ETDRS 10–20), from 33% to 100% (ETDRS 35) and from 33 to 100% (ETDRS 43–47) (Table 3).

All six eyes with CI-DMO at the end of the 3-year period of follow-up, showed marked increases in extracellular retinal fluid, represented by increased LOR ratio values **≥**1SD (6/6, 100%).

In the eyes with subclinical CI-DMO and CI-DMO the LOR values showed marked fluctuations between visits, with the presence of these fluctuations being associated with vision loss (Table 3).

When considering the subclinical CI-DMO and CI-DMO eyes with vision loss over the 3-year period, 9 out of 11 (82%) show multiple LOR fluctuations, while 8 out of 12 (67%) eyes with vision gain had one or less LOR fluctuations (*p* value = 0.047).

## Discussion

The results here reported confirm that eyes in the initial stages of diabetic retinopathy in T2D individuals show evidence of CI-DMO identified by OCT in a relatively small number of eyes (~10%) and that CI-DMO show similar prevalence in eyes with different grades of ETDRS severity below level 47. In summary, CI-DMO, i.e., ≥2 SD CRT increase, can occur very early in the disease process and is, in general, not apparently related to DR severity.

The ETDRS identified clinically significant macular oedema as a clear indication for treatment [8]. However, more clinically relevant today, is the classification of diabetic macular oedema (DMO) as either CI-DMO and non-CI-DMO [7, 16]. Central involvement with concurrent thickening affecting the 1 mm diameter central subfield thickness is most accurately determined by OCT and it is also independent of individual doctor perception. It should be realised that OCT depends on patient fixation for accurate measurements and technician ability.

In cases of CI-DMO, there are two key subcategories: those with preserved best-corrected visual acuity (>20/32) and those with associated vision loss [7]. These two subcategories must be considered when evaluating DMO and may indicate not only different treatment approaches but also different associated risk factors, namely association with retinal ischaemia. When considering DMO we have shown previously that systemic markers are not as good predictors as ocular imaging markers [17].

In this study, we followed for a period of 3-years, eyes categorised as minimal, mild and moderate NPDR using seven-field ETDRS grading. Macular oedema, subclinical or clinical, identified respectively by increased CRT (≥1SD and ≥2SD) did not show significant progression during the 3-year period of follow-up and did not show an association with retinopathy severity worsening. It should be realised, however, that vision may be preserved until severe ischaemia is present [18].

It is of major relevance to characterise progression of macular oedema in the initial stages of DR in order to identify opportunities for early intervention before disease progression and vision loss. Subclinical CI-DMO has been proposed as a predictor for development of CI-DMO and it would be of major interest to use OCT as an objective measurement of CI-DMO and a reference to identify DMO needing close surveillance to guarantee timely treatment.

Our study shows that subclinical CI-DMO and CI-DMO can occur at any ETDRS level, with similar prevalence, and that subclinical CI-DMO is not predictive of evolution to CI-DMO in a 3-year period.

It is apparent from this study, that subclinical CI-DMO does not inexorably progress over time scales of 1–3 years and, furthermore, a fraction of these eyes spontaneously improved. We observed, however, in the 3-year period of follow-up, that some eyes with increased CRT show a progressive increase in LOR values, indicating a progressive increase in retinal extracellular fluid accumulation when there is a chronic and prolonged situation of increased retinal thickness, representing oedema. Furthermore, eyes with CI-DMO and visual loss show a clear association with increased retinal fluid accumulation, demonstrated by increased LOR ratio values, whereas eyes with CI-DMO and no vision loss did not show increases in extracellular retinal fluid. Abnormal accumulation of retinal extracellular fluid appears to be a good indicator for the need for close monitoring with potential for early treatment. Further investigations such as fundus fluorescein angiography may be utilised to assess the ischaemic element of macular oedema.

Recent reports have called attention to the association of vision loss to fluctuations in CRT in eyes with macular oedema [19, 20]. We confirmed that eyes with subclinical CI-DMO and CI-DMO showing marked fluctuations in LOR values, representing retinal extracellular fluid accumulation, are associated with vision loss. The presence of increased abnormal retinal extracellular fluid offers an explanation for the occurrence of frequent fluctuations as extracellular fluid would be expected to change more easily than intracellular fluid. The presence of abnormally increased extracellular fluid may manifest itself by wider fluctuations in LOR and CRT values and serve as a sign more extensive damage of the retina.

A limitation of this study is the number of eyes included in the study, which may introduce a bias in our findings. However, the distribution of eyes with ETDRS severity grades in the initial stages of DR relatively is well balanced and the CRT and LOR ratio measurements were performed in retinas that remained structurally preserved with no evidence of cystoid changes. None of the patients showed retinal structural changes such as disorganisation of retinal inner layers or full disruption of the ellipsoid zone [21, 22]. Of special value, the fact that a strict quality check was performed by a masked grader.

In conclusion, eyes from T2D patients with minimal, mild and moderate NPDR followed for a period of a 3-years, with annual visits, show evidence of subclinical CI-DMO and CI-DMO with and without visual loss in a relatively small percentage of cases. The changes in CRT and in LOR ratios do not appear to be directly associated with changes in VD (ischaemia) or GCL + IPL thinning (neurodegeneration). Furthermore, subclinical CI-DMO did not appear to be a predictor of development of CI-DMO in a period of 3 years. There was however a progressive increase in retinal extracellular fluid registered in the eyes with subclinical CI-DMO and CI-DMO with vision loss, suggesting a relevant role for retinal extracellular fluid accumulation and chronicity in the development of vision loss in the initial stages of macular oedema.

Identification and quantification of abnormal extracellular fluid accumulation in the diabetic retina and its location in the different layers of the retina may explain progression of macular oedema and associated vision loss.

### Summary

#### What was known before

- Central-involving macular oedema (CI-DMO) is objectively determined by OCT and is independent of individual doctor perception.
- There are two subtypes of individuals with CI-DMO: those which preserve best-corrected visual acuity and those associated with visual loss.
- Macular oedema can occur in any ETDRS severity grade.

#### What this study adds

- Confirmation that subclinical and clinical CI-DMO in NPDR occurs independently of severity grades of the retinopathy.
- Eyes with subclinical and clinical CI-DMO followed for a period of 3-years show a progressive increase in retinal extracellular fluid.
- The eyes with CI-DMO and increased retinal extracellular fluid are associated with vision loss.

## Data Availability

Data will be available upon request to the correspondent author.
